# Evaluation of Cracking Resistance of SMA-13 Hot Recycling Asphalt Mixtures Reinforced by Basalt Fiber

**DOI:** 10.3390/ma17081762

**Published:** 2024-04-11

**Authors:** Yu Zhang, Yao Zhang, Bo Li, Aihong Kang, Yu Wang

**Affiliations:** 1 College of Civil Science and Engineering, Yangzhou University, Yangzhou 225127, China; zy502499180@163.com (Y.Z.); yaozhang@yzu.edu.cn (Y.Z.); aihongkang@yzu.edu.cn (A.K.); wangyu@163.com (Y.W.); 2 Research Center for Basalt Fiber Composite Construction Materials, Yangzhou 225127, China

**Keywords:** asphalt mixture, hot recycling, cracking resistance, basalt fiber, low-temperature creep

## Abstract

In the context of green and low-carbon development, energy saving, and emission reduction, hot recycling technology (RT) has been researched, which is divided into hot central plant RT and hot in-place RT. However, due to the aged asphalt binders, the shortcomings of hot recycled asphalt mixtures have become apparent, as in comparison to new asphalt mixtures, their resistance to cracking was inferior and the cracking resistance deteriorated more rapidly. Therefore, it was very necessary to focus on the improvement of crack resistance of hot recycled asphalt mixtures. Basalt fiber has been proved to be able to effectively improve the comprehensive road performance of new asphalt mixtures. Therefore, this paper introduced basalt fiber to hot central plant recycled and hot in-place recycled asphalt mixtures, in order to improve the crack resistance of asphalt as a new type of fiber stabilizer. Firstly, six types of SMA-13 fiber asphalt mixtures were designed and prepared, i.e., hot mixtures with basalt fiber or lignin fiber, hot central plant recycled mixtures with basalt fiber or lignin fiber, and hot in-place recycled mixtures with basalt fiber or lignin fiber. Secondly, the trabecular bending test, low-temperature creep test, semi-circular bending test, and IDEAL-CT were used to comparatively study the changing patterns of low and intermediate temperature cracking resistance of hot recycled mixtures with conventional lignin fibers or basalt fibers. Finally, Pearson’s correlation coefficient was used to analyze the correlation of the different cracking resistance indicators. The results show that the low and intermediate temperature cracking resistance of hot central plant recycled mixtures increased by 45.6% (dissipative energy ratio, *W_d_*/*W_s_*) and 74.8% (flexibility index, FI), respectively. And the corresponding cracking resistance of hot in-place recycled mixture increased by 105.4% (*W_d_*/*W_s_*) and 55.7% (FI). The trabecular bending test was more suitable for testing the low-temperature cracking resistance of hot recycled asphalt mixtures, while the IDEAL-CT was more suitable for testing the intermediate-temperature cracking resistance. The results can provide useful references for the utilization of basalt fiber in the hot recycling of SMA-13 asphalt mixtures.

## 1. Introduction

Asphalt pavement hot recycling technologies, encompassing hot central plant recycling and hot in-place recycling, have gained increasing popularity due to their numerous advantages. These advantages include rapid execution, cost-effectiveness, and resource conservation. By utilizing these technologies, the efficiency of resource utilization could be significantly enhanced, thereby promoting green and low-carbon development [1,2,3]. In Europe, more than 70% of the annual recycled asphalt pavement material (RAP) is recycled [4]. According to statistics from the Federal Highway Administration (FHWA) and the National Asphalt Pavement Association (NAPA), the majority of recycled waste asphalt pavement materials in the United States are utilized in the production of hot-mix asphalt mixtures, representing a significant proportion ranging from 83 to 95 percent of the total recycled materials [5]. Comparatively, in China, the recycling rate of asphalt pavement materials stands at 30 percent, indicating a notable gap in the utilization of recycled asphalt materials between the two countries [6].

However, performance deterioration of hot recycled asphalt mixtures, especially the insufficiency of cracking resistance, has been noticed, though the essential performance meets the requirements [7,8,9]. Through low-temperature crack resistance testing, researchers have discovered that a hot recycled asphalt mixture exhibits increased viscosity and brittleness compared to the original asphalt mixture. This finding suggests that the recycled material may have altered the physical properties of the asphalt, potentially affecting its performance and durability under low-temperature conditions [10,11,12]. The interface between a new asphalt mixture and a reclaimed asphalt mixture will increase the concentrated stress, resulting in a tendency of cracking in low-temperature environments [13,14,15]. Indeed, various methods have been employed to assess the cracking resistance of recycled asphalt mixtures. T. Mandal et al. conducted measurements to assess the impact of binder replacement from recycled asphalt pavement, binder modification, and low-temperature binder grade on the DCT test results. However, it was discovered that the significance of these factors was insufficient to account for the full range of DCT response variables [16]. S. Xiang et al. conducted an evaluation of the impact of reclaimed asphalt pavement (RAP) on the cracking resistance of asphalt mixtures using the SCB test. Their findings indicate that, while RAP generally enhances the SCB tensile strength of the mixtures, it significantly reduces their post-failure tenacity. Furthermore, the J-integral (fracture criterion in elastoplastic fracture mechanics) of the asphalt mixture decreased with the addition of RAP, resulting in a weakened cracking resistance [17]. Z. Fu et al. conducted a comparison between IDEAL-CT results and field cracking data gathered from various sources, including the Federal Highway Administration’s accelerated load facility, Texas SH15 and SH62, as well as MnROAD. The IDEAL-CT indicators demonstrated a strong correlation with field performance in respect to fatigue, reflective and thermal cracking [18]. To improve the low-temperature cracking resistance of recycled asphalt mixtures, H. Jing et al. determined the optimum design of recycled asphalt mixtures through the trabecular bending test [19]. K. Moon et al. conducted a study on the impact of incorporating both reclaimed asphalt pavement (RAP) and recycled asphalt shingles (RAS) into virgin asphalt mixtures through a simple low-temperature creep test utilizing asphalt mixture beams. The findings revealed that most mixtures formulated with combinations of RAP and RAS exhibited comparable performance to standard mixtures at low temperatures [20].

Scholars are also searching for measures to improve the cracking resistance of hot recycled mixtures. The normally used ones include improving the mixing process of hot recycled asphalt mixtures [21]; using external mixing additives [22] such as glass fibers, polyester fibers, high modulus agents, rutting resistance agents, etc.; adding recycled plastics [23]; using rejuvenators [17]; and so on.

Currently, basalt fiber is extensively employed to boost the overall performance of asphalt mixtures, resulting in a notable enhancement in crack resistance [24,25,26,27]. Z. Yao et al. examined a basalt fiber asphalt mixture through mid- to low-temperature (25 °C and −10 °C) indoor cracking tests, including the trabecular bending test, IDEAL-CT test, and SCB test. They delved into the correlation between crack resistance and morphological parameters, and further explored the impact of fiber length on the fracture characteristics of the basalt fiber asphalt mixture [24]. By incorporating basalt fibers into asphalt mixtures, H. Ying et al. discovered that it effectively enhanced the performance characteristics of the mixture, including its cracking resistance, rutting resistance, and fatigue resistance [28]. The potential for improved cracking resistance in hot recycled asphalt mixtures was indicated by the inference that their performance may be enhanced through the incorporation of basalt fibers.

The purpose of this study is to explore the impact of basalt fibers on the cracking resistance performance of both hot central plant recycled and hot in-place recycled asphalt mixtures. The intended target is the gradation of Stone Matrix Asphalt with a nominal maximum aggregate size (NMAS) of 13.2 mm, referred to as SMA-13. Trabecular bending tests and creep testes are used to evaluate the low-temperature performance, while semi-circular bending tests and IDEAL cracking tests are conducted to test the intermediate-temperature cracking properties.

## 2. Materials and Methods

### 2.1. Materials

#### 2.1.1. Reclaimed Asphalt Pavement (RAP)

The RAP used in this paper was taken from the Wuxi section of the Shanghai–Nanjing expressway. The upper layer was with the gradation of SMA-13. The old asphalt was extracted according to the specification JTG 5142-2019 [29], including extraction, high-speed centrifugation, and rotary evaporation. Then, the extracted old asphalt was tested for penetration, softening point, ductility, and viscosity. The test results are shown in Table 1.

In addition, the asphalt content and mineral gradation of the old pavement were determined according to the methods specified in JTG E20-2011 (T0722-1993) and (T0725-2000). The asphalt content of RAP was 5.48%, and the mineral gradation is shown in Table 2.

#### 2.1.2. Rejuvenating Agent

The rejuvenating agent RA-102, manufactured by Subote New Materials Co., (Yangzhou, China) was employed in this study. The specific technical indexes pertaining to RA-102 are detailed in Table 3.

#### 2.1.3. New Aggregates and New Asphalt

For the aggregates, basaltic coarse and fine aggregates were chosen, and limestone mineral powder was selected as the filler. All the aggregates and fillers underwent testing according to the corresponding requirements of the Test Procedure for Aggregates in Highway Engineering (JTG E42) [32]. The results of these tests were found to be compliant with the specified standards.

The SBS-modified asphalt with a PG grade of 76-22 was chosen in this study. It had a needle penetration of 54 dmm, a softening point of 80 °C, and a rotational viscosity of 2.35 Pa·s at 135 °C. The corresponding requirements of the test specification were met by the test results.

#### 2.1.4. Basalt Fiber

The basalt fiber utilized in this study was sourced from Jiangsu Tianlong Basalt Continuous Fiber Co., Ltd. (Yizheng, China), and its technical properties are presented in Table 4.

#### 2.1.5. Lignin Fiber

Hot recycled asphalt mixtures with lignin fiber were prepared and utilized as comparison groups to those containing basalt fiber. The technical properties of the lignin fiber are detailed in Table 5.

### 2.2. Mixture Design

#### 2.2.1. Gradation

**Figure 1:**
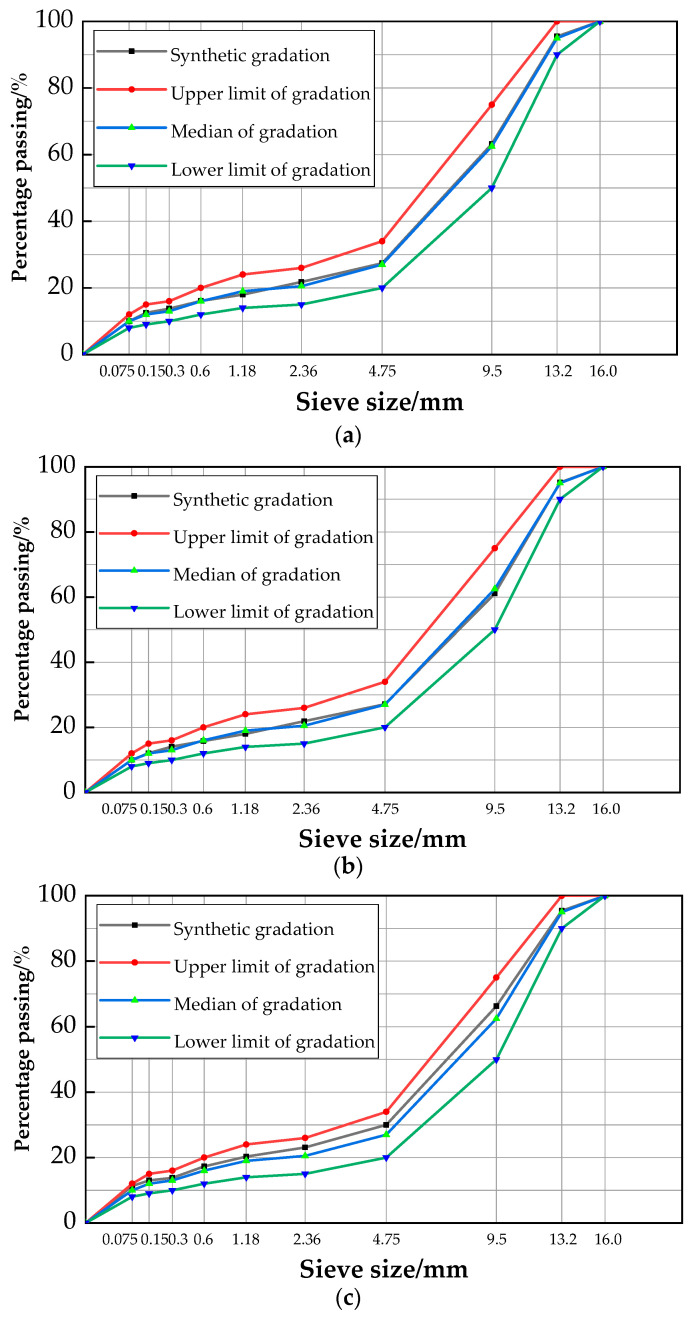
Synthetic gradation: (**a**) hot central plant recycled asphalt mixture; (**b**) hot in-place recycled asphalt mixture; (**c**) hot mix asphalt mixture.

Three SMA-13 asphalt mixture types were studied: (1) hot mix (0% RAP), (2) hot central plant mix (30% RAP), and (3) hot in-place mix (80% RAP). For each type of mixture, two sets of samples were fabricated either with lignin fiber or with basalt fiber. The synthetic gradation of the new asphalt mixes, hot in-central plant RT mixes, and hot in place RT mixes are shown in Figure 1.

#### 2.2.2. Dosage of Rejuvenating Agent

To produce the recycled asphalt, dosages of the rejuvenating agent were set at 4%, 6%, 8%, and 10% by weight of old asphalt. Conventional performance evaluations were conducted through penetration, softening point, and ductility tests. The test results, presented in Table 6, indicated that when the rejuvenating agent dosage reached 6%, the penetration and softening point of the recycled asphalt nearly reached comparable levels to that of new asphalt, while the ductility of the recycled asphalt recovered to a certain extent. Based on these findings, a rejuvenating agent dosage of 6% was chosen for this study.

#### 2.2.3. Optimum Oil/Aggregate Ratio

Optimum oil/aggregate ratio of each type mixture was determined according to JTG E20. The final optimum asphalt content and the corresponding volumetric parameters are summarized in Table 7. For both hot in-place recycled mixes and hot central plant recycled mixes, the oil/aggregate ratio of basalt fiber mixtures was 0.2 percentage points less than that of the lignin fiber mixtures. As far as hot in-place recycled mixtures were concerned, the oil/aggregate ratio was the same for both mixtures with basalt fiber or lignin fiber.

According to the specifications in JTG E20-2011, T0732, and T0733, Schellenberg leakage tests and Fort Kentucky flyaway tests were conducted to verify whether the amount of asphalt in the mixture was excessive and whether the cohesion between aggregate and asphalt was strong enough. The asphalt content of each mixture type was deemed adequate based on the results of the tests conducted on the six types of mixtures, which are summarized in Table 8. These findings were in compliance with the established requirements.

#### 2.2.4. Preparation of Recycled Asphalt Mixture

**Figure 2:**
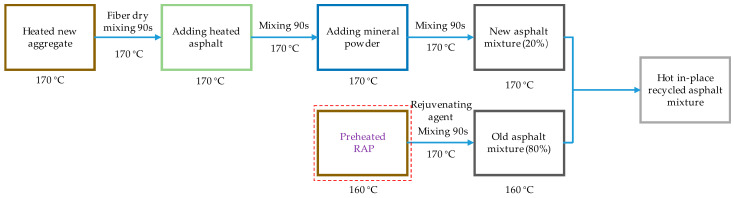
Indoor simulation preparation process of hot in-place recycled mixtures.

**Figure 3:**
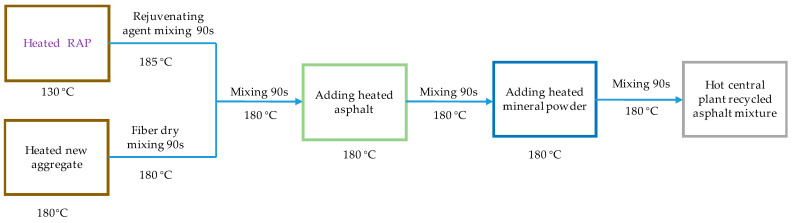
Indoor simulation preparation process of hot central plant recycled mixtures.

Both hot central plant recycled and hot in-place recycled asphalt mixtures were prepared according the corresponding construction processes, as shown in Figure 2 and Figure 3. The new fibers, either lignin fibers or basalt fibers, were mixed with the heated new aggregates, which is called the dry mixing process. And then, the recycled mixtures could be fabricated by mixing the new asphalt mixture and preheated RAP. It can be noticed from Figure 2 and Figure 3 that the heating temperatures were obviously different. The heating temperatures for the new asphalt mixture and RAP were set at 180 °C and 130 °C, respectively, for the hot central plant recycled mixture. For the hot in-place recycled mixture, the corresponding heating temperatures were 170 °C and 160 °C.

### 2.3. Test Method

#### 2.3.1. Trabecular Bending Test

**Figure 4:**
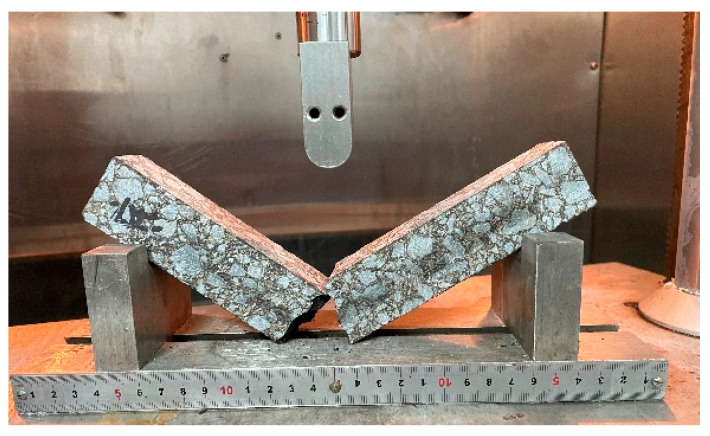
Illustration of trabecular bending test.

In accordance with the specifications in JTG E20-2011, the trabecular bending test was conducted to analyze the low-temperature cracking resistance of recycled asphalt mixtures. Prismatic beam samples measuring 250 mm × 30 mm × 35 mm were prepared for the testing. Four duplicate samples per group were tested at a temperature of −10 °C and a loading rate of 50 mm/min. The bending tensile strength (*R_B_*/MPa), maximum failure strain (ε_B_/με), and stiffness modulus (S_B_/MPa) were then calculated using Equations (1)–(3). The test procedure is shown in Figure 4.

(1) $$R_{B}=\frac{3\times L\times P_{B}}{2\times b\times h^{2}}$$

(2) $$\varepsilon_{B}=\frac{6\times h\times d}{L^{2}}$$

(3) $$S_{B}=\frac{R_{B}}{\varepsilon_{B}}$$

where *b* is the width of the specimen, mm; *h* is the height of specimen, mm; *L* is the length of the specimen, mm; *P_B_* is the maximum load, N; and *d* is the span deflection when the specimen failed, mm.

#### 2.3.2. Low-Temperature Creep Test

The low-temperature bending creep test, as stipulated in the JTG E20-2011 specification, was an effective means to evaluate the low-temperature performance of asphalt mixtures, taking into account factors like the creep rate and dissipation energy. The specimens used for this test were prism-shaped, with dimensions of 250 mm ± 2 mm in length, 30 mm ± 2 mm in width, and 35 mm ± 2 mm in height.

**Figure 5:**
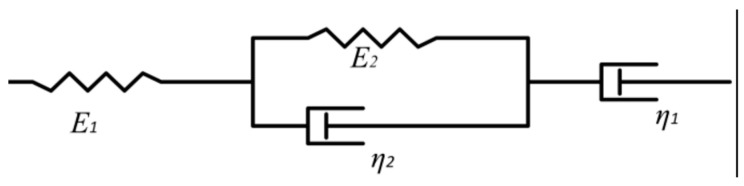
Illustration of the Burgers model.

Due to the typical viscoelastic characteristics, the Burgers model was usually used to characterize the mechanical properties of the asphalt mixtures. The model was composed of the Maxwell model and the Kelvin model. The model consisted of two elastic elements (*E*_1_, *E*_2_) and two viscous elements (*η*_1_, *η*_2_), as shown in Figure 5.

The four-parameter creep equation of the Burgers model can be expressed by Equation (4).

(4) $$\varepsilon (t)=\sigma_{0}\left[\frac{1}{E_{1}}+\frac{t}{\eta_{1}}+\frac{1}{E_{2}}(1-e^{-\frac{E_{2}}{\eta_{2}}t})\right]$$

where *ε*(*t*) is the bending tensile strain of the beam bottom; *σ*_0_ is the creep bending tensile stress of specimen, MPa; *t* is the load time, s; *E*_1_, *E*_2_ are the elastic moduli; and *η*_1_, *η*_2_ are the coefficients of viscosity.

The optimal solution of the four parameters in the Burgers model could be obtained by nonlinear fitting using the global optimization algorithm through the changeable data of mid-span deflection with time collected by the test. Once the optimal solution for the four parameters in the Burgers model was obtained, they were utilized to calculate the dissipation energy (*W_d_*), storage energy (*W_s_*), bending stiffness modulus *S*(*t*), and creep rate *m*(*t*) for each mixture type. These calculations served to characterize the low-temperature performance of each mixture. The essence of each element in the Burgers model was the spring and damper, corresponding to the storage energy and dissipation energy, respectively. According to the literature [20], the dissipative energy ratio of *W_d_*/*W_s_* can accurately represent the low-temperature performance of the material, with a higher value indicating better low-temperature performance. Moreover, contradictory phenomena frequently arose when relying solely on a single bending stiffness modulus and creep rate. However, by utilizing the creep rate per unit stiffness, *m*(*t*)/*S*(*t*), the contradictory phenomenon could be effectively avoided, thereby accurately reflecting the strengths and weaknesses of the material’s low-temperature performance [21]. Specifically, a higher value of *m*(*t*)/*S*(*t*) indicates better low-temperature performance. The specific calculation equations for *m*(*t*)/*S*(*t*), *W_d_*, and *W_s_* are presented in Equations (5), (6), and (7), respectively.

(5) $$\frac{m(t)}{S(t)}=\left(\frac{1}{\eta_{1}}+\frac{1}{\eta_{2}}e^{-\frac{E_{2}}{\eta_{2}}t}\right)$$

(6) $$W_{d}(t)={\sigma_{0}}^{2}\left[\frac{t}{\eta_{1}}+\frac{1}{2E_{2}}(1-e^{-\frac{2E_{2}}{\eta_{2}}t})\right]$$

(7) $$W_{s}(t)={\sigma_{0}}^{2}\left[\frac{1}{E_{1}}+\frac{1}{2E_{2}}(1-2e^{-\frac{E_{2}}{\eta_{2}}t}+e^{-\frac{2E_{2}}{\eta_{2}}t})\right]$$

where *W_d_* is the stored energy; *W*_s_ is the dissipative energy; *S*(*t*) is the bending modulus of strength; and *m*(*t*) is the creep rate.

#### 2.3.3. Semi-Circular Bending Test

To assess the intermediate anti-cracking capability of asphalt mixtures, the semi-circular bending (SCB) test was conducted in accordance with the standard test method outlined in AASHTO TP 124-16 [37]. During this test, the load and displacement curves were carefully recorded. The fracture work generated throughout the cracking process was represented by the area enclosed by these curves. The fracture energy, defined by Equations (8)–(10), was calculated as the ratio of the fracture work to the fracture area. A higher value of fracture energy indicates superior anti-cracking performance. For accurate results, four duplicate samples were used for each group.

(8) $$G_{f}=\frac{W_{f}}{{\mathrm{Area}}_{\mathrm{lig}}}\times 10^{6}$$

(9) $$W_{f}=\int Pdu$$

(10) $$A_{lig}=(r-a)t$$

where *G_f_* is the fracture energy, J/m^2^; *W_f_* is the fracture work, J; *A_lig_* is the area of fracture area, mm^2^; *r* − *a* is the length of the fracture zone; and *t* is the thickness of specimen, mm.

**Figure 6:**
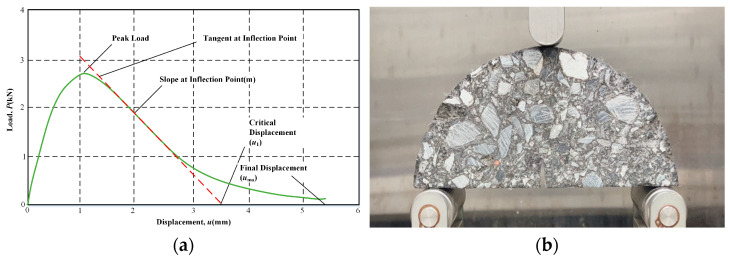
Illustration of semi-circular bending test: (**a**) load (P) vs. displacement (u) curve; (**b**) test procedure.

The flexibility index (FI) was introduced as a metric to reflect and characterize the crack propagation rate, as depicted in Figure 6a. Equation (11) provides the mathematical expression for this FI index. Furthermore, Figure 6b outlines the test procedure.

(11) $$FI=\frac{G_{f}}{\left|m\right|}\times A$$

where *FI* is the flexibility index; |*m*| is the absolute value of the post-peak slope, kN/mm; and *A* is the unit conversion coefficient, which is 0.01.

#### 2.3.4. IDEAL Cracking Test

**Figure 7:**
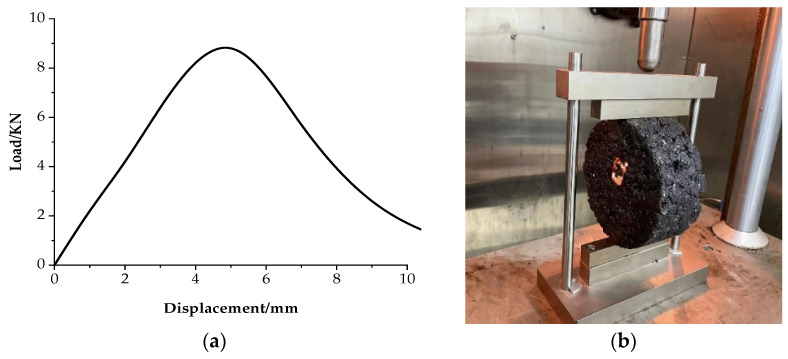
The test procedure of IDEAL-CT: (**a**) test conditions; (**b**) specimen and fixture.

The IDEAL-CT test was one of the methods employed to assess the crack resistance of asphalt mixtures. In this test, a cylindrical asphalt mixture specimen measuring 62 mm in height and 150 mm in diameter was used [18]. The CT_index_ proposed in this test served as a metric to evaluate the anti-cracking performance of the asphalt mixtures. This index reflected the asphalt mixtures’ resistance to crack propagation, and its calculation formulas are detailed in Equations (12) and (13). The test procedure is illustrated in Figure 7.

(12) $$\left|m_{75}\right|=\left|\left(p_{85}-p_{65}\right)/\left(l_{85}-l_{65}\right)\right|$$

(13) $$CT_{\mathrm{Index}}=\frac{G_{f}}{\left|m_{75}\right|}\times (\frac{l_{75}}{D})$$

where *CT*_index_ is the cracking test index; *t* is the specimen thickness, mm; *G_f_* is the fracture energy, J/m^2^; *m*_75_ is the absolute value of the slope at 75% peak in the post-peak section; *l*_75_ is the displacement at 75% of the peak value in the post-peak section, mm; and *D* is the specimen diameter, mm.

## 3. Results and Discussion

### 3.1. Results of Trabecular Bending Test

**Figure 8:**
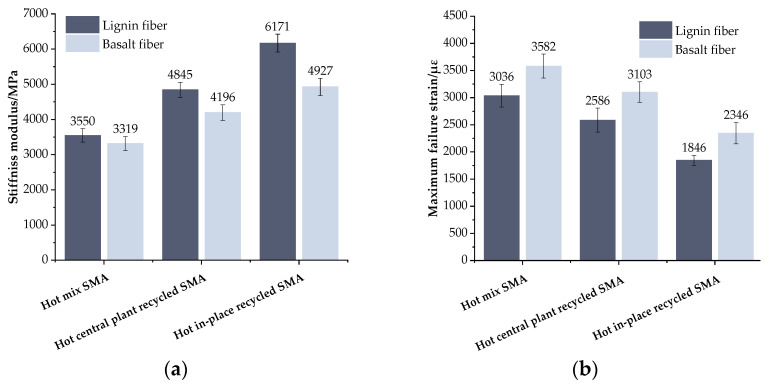
Results of trabecular bending test: (**a**) stiffness modulus; (**b**) failure stain.

The results of the stiffness modulus and maximum failure strain obtained from the trabecular bending tests are graphically represented in Figure 8. It is evident from Figure 8 that, for a given fiber type, as the RAP content increases, the stiffness modulus of the recycled asphalt mixture also increases, whereas the maximum failure strain exhibits a decreasing trend. Especially in the case of the hot in-place recycled mixture with 80% RAP, when the conventional lignin fiber was used, the maximum failure strain only reached 1846 με, which was far less than the specification requirement of not less than 2500 με. This means that the addition of a large content of RAP would have an adverse effect on the low-temperature performance of the recycled mixture. This is because the aromatics and saturates were oxidized into resins and asphaltenes, resulting in the more brittle and less tough characteristics of the aged asphalt in RAP [38]. Even if the incorporation of rejuvenating agent softened the aged asphalt to a certain extent, its low-temperature performance still presents a certain gap to the specification requirements.

When the mixture type was the same, the stiffness modulus of the recycled asphalt mixture reinforced by basalt fiber decreased greatly, while the maximum failure strain was significantly improved compared with that of the lignin fiber recycled one. When the RAP content was 0%, 30%, and 80%, the maximum failure strain of the corresponding mixture increased by 18%, 20%, and 27%, respectively. This observation can be primarily attributed to the inherent characteristics of basalt fiber, which possesses high strength and a high modulus [17]. At low temperatures, basalt fiber is closely combined with asphalt and has strong integrity, which enhances the elasticity of the basalt fiber recycled mixture, thus improving its anti-cracking ability at low temperatures.

### 3.2. Results of Low-Temperature Creep Test

**Figure 9:**
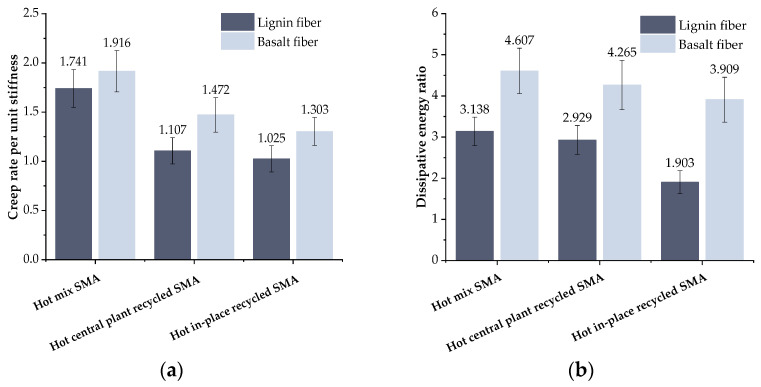
Results of low-temperature creep test: (**a**) creep rate per unit stiffness (*m*(*t*)/*S*(*t*)); (**b**) dissipative energy ratio (*W_d_*/*W_s_*).

According to the results from the low-temperature bending creep test, the ratio of *W_d_*/*W_s_* and *m*(*t*)/*S*(*t*) at *t* = 3000 s was calculated, and the calculated results of *W_d_*/*W_s_* and *m*(*t*)/*S*(*t*) are presented in Figure 9. As shown in Figure 9, whether basalt fiber or lignin fiber was used, the *W_d_*/*W_s_* and *m*(*t*)/*S*(*t*) of recycled mixtures show a downward trend with the increase in RAP content, indicating that RAP weakens the stress relaxation ability of the recycled mixture and makes it difficult to dissipate the cumulative stress timely, resulting in the inferior low-temperature crack resistance. These results are also consistent with the test results of the trabecular bending test.

When comparing the mixture types, it was observed that the basalt fiber recycled asphalt mixture exhibited significantly improved values for *m*(*t*)/*S*(*t*) and *W_d_*/*W_s_* compared to the lignin fiber recycled asphalt mixture. Specifically, at a RAP content of 30%, the basalt fiber recycled mixture demonstrated an increase of 33% in *m*(*t*)/*S*(*t*) and 45.6% in *W_d_*/*W_s_*. When the content of RAP was 80%, the *m*(*t*)/*S*(*t*)and *W_d_*/*W_s_* of the basalt fiber recycled mixture increased by 27.1% and 105.4%, respectively. This was consistent with the findings of L. Co et al. [39] who showed that the effect of basalt fibers was still relatively significant even in the case of different types of asphalt mixtures. According to the composite material theory, when basalt fiber is added into the recycled asphalt mixture, a three dimensional network structure will form in the mixture. A large number of new interfaces will also be generated, which can reduce the temperature sensitivity of asphalt [39]. Meanwhile, the network structure formed in the mixture could play a role in dissipating stress concentration to a certain extent [40], thereby improving the low-temperature cracking resistance of the mixture.

### 3.3. Results of Semi-Circular Bending Test

**Figure 10:**
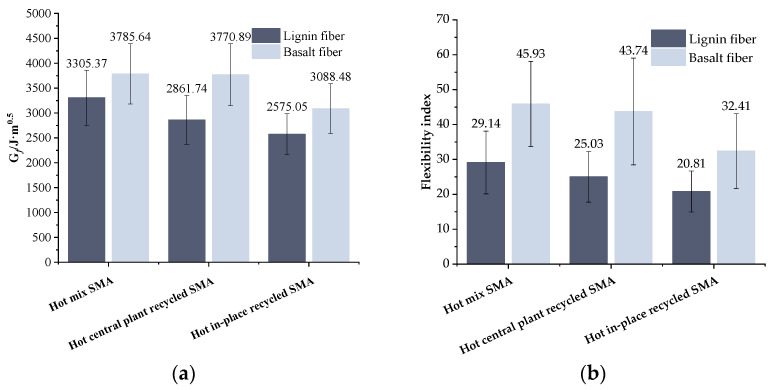
Results of semi-circular bending test: (**a**) fracture energy (G*_f_*); (**b**) flexibility index (FI) of recycled asphalt mixture.

Figure 10 presents the results obtained from the semi-circular bending tests. A careful analysis of Figure 10 indicates that, as the RAP content increased, the fracture energy (G*_f_*) and flexibility index (FI) of the recycled asphalt mixture decreased for a particular fiber type. This observation implies that RAP had a detrimental effect on the recycled mixtures’ resistance to cracking at intermediate temperatures. This is because the old asphalt becomes hard and brittle after aging, which reduces the overall relaxation ability of the recycled asphalt mixture. Therefore, with a high RAP content, the asphalt pavement is more prone to cracking. When the mixture type was the same, compared with the lignin fiber recycled asphalt mixture, the G*_f_* of the basalt fiber recycled asphalt mixture was greatly improved. At 0%, 30%, and 80% RAP content, the G*_f_* of basalt fiber mixture increased by 14.5%, 31.8%, and 19.9%, respectively, indicating that the mixture could absorb more cracking energy after adding basalt fiber. The FI of the corresponding mixture increased by 50.8%, 74.8%, and 55.7%, respectively, indicating that basalt fiber could delay the propagation of cracks. This trend was consistent with the findings of W. Bang et al. [41]. This is due to the load dispersion and transmission ability of basalt fiber, which can reduce the stress concentration, thereby improving the anti-crack propagation ability of the mixture.

### 3.4. Results of IDEAL-CT Cracking Test

**Figure 11:**
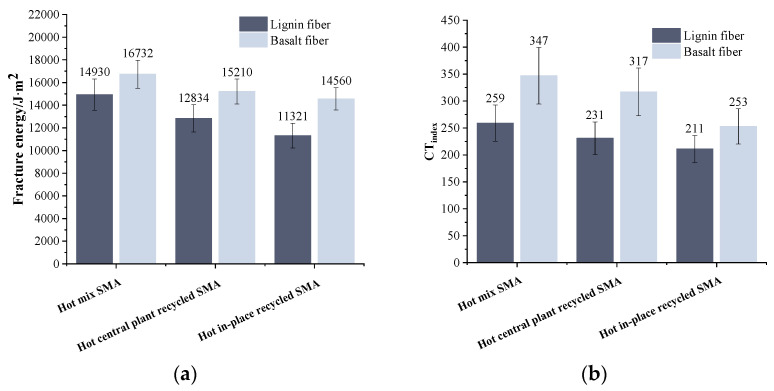
Results of IDEAL-CT cracking test: (**a**) fracture energy (G*_f_*_0_); (**b**) crack resistance index (CT_index_).

The results of the IDEAL-CT cracking tests are shown in Figure 11. It is evident from Figure 11 that as the RAP content increased, the fracture energy (G*_f_*_0_) and cracking index of asphalt mixtures with the same fiber type displayed a downward trend. This trend suggests that the intermediate-temperature crack resistance of the mixture is weakened by RAP, making the asphalt mixture more prone to brittleness. When the mixture type was the same, compared with the lignin fiber recycled asphalt mixture, the fracture energy (G*_f_*_0_) and CT_index_ of the basalt fiber recycled asphalt mixture were significantly improved. As for the hot central plant recycled mixture (30% RAP) the G*_f_*_0_ and CT_index_ increased by 18.5% and 37.2%, respectively. In terms of the hot in-place recycled one (80% RAP), the G*_f_*_0_ and CT_index_ increased by 28.6% and 19.9%, respectively. This infers that basalt fiber presents superior enhancing ability to the crack resistance of recycled asphalt mixture than lignin fiber.

### 3.5. Correlation Analysis

Differences in the crack resistance of hot central plant recycled and hot in-place recycled asphalt mixtures at low and intermediate temperatures have been demonstrated through the preceding discussions. To gain a deeper understanding of how the crack resistance of hot recycled asphalt mixtures depended on various fibers, a correlation analysis of mixture detection indexes was conducted. In this study, Pearson’s correlation coefficient was used to investigate the correlation between the results of each crack resistance index, with the mix type (RAP content) as the variable. Through IBM SPSS Statistics 27.0, the anti-cracking indexes (maximum failure strain, G*_f_*, FI, G*_f_*_0_, CT_index_, *W_d_*/*W_s_*, and *m*(*t*)/*S*(*t*)) were imported into the SPSS software, and the quotient of the product of covariance and standard deviation between variables was calculated and defined as Pearson’s correlation coefficient ‘r’. The calculation formula is expressed by Equation (14). The value range of Pearson’s correlation coefficient, denoted as ‘r’, lies between −1 and 1. The degrees and interpretation of Pearson’s correlation coefficient are shown in Table 9 [42].

(14) $$r=\frac{\sum_{i=1}^{n}\left(x_{i}-\bar{X})(y_{i}-\bar{Y}\right)}{\sqrt{\sum_{i=1}^{n}{\left(x_{i}-\bar{X}\right)}^{2}}\sqrt{\sum_{i=1}^{n}{\left(x_{i}-\bar{Y}\right)}^{2}}}$$

where x_i_, y_i_ are the corresponding cracking indicators; $\bar{X},\bar{Y}$ are the average values of the crack resistance index; and r is Pearson’s correlation coefficient.

**Figure 12:**
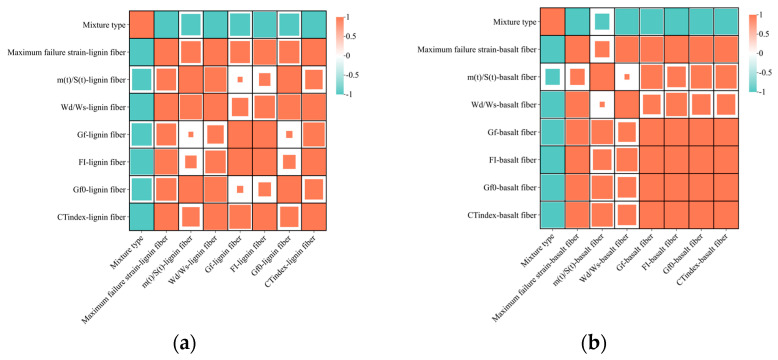
Correlation analysis of indicators: (**a**) lignin fiber hot recycled asphalt mixture; (**b**) basalt fiber hot recycled asphalt mixture.

The horizontal and vertical coordinates in Figure 12 show the test metrics for each cracking test. Figure 12a displays the correlation of each cracking indicator for the lignin fiber asphalt mixture, while Figure 12b presents the corresponding correlation for the basalt fiber asphalt mixture. In the visualization of the correlation matrix, a positive correlation is represented by orange. Conversely, green represents a negative correlation. The size of the square within the matrix corresponds to the degree of correlation between the respective variables x_i_ and y_i_. The greater the correlation, the larger the area of the square, and vice versa. According to Figure 12, comparing the indexes of low-temperature cracking resistance (maximum failure strain, *W_d_*/*W_s_*, and *m*(*t*)/*S*(*t*)), the correlation between maximum failure strain and other indicators was better. From a mathematical perspective, the trabecular bending test is more effective in evaluating the low-temperature cracking resistance of hot recycled asphalt mixtures. When comparing indices of mid-temperature cracking resistance such as Gf, FI, G*_f_*_0_, and CT_index_, it was observed that the correlation between G*_f_*_0_ (and/or CT_index_) and the other indicators was stronger. Therefore, the IDEAL-CT test is more suitable for assessing the mid-temperature cracking resistance of hot recycled asphalt mixtures.

## 4. Conclusions

This study examined the impact of basalt fiber on the cracking resistance of hot recycled asphalt mixtures through various low- and intermediate-temperature (−10 °C and 25 °C) cracking tests. Based on these tests, the following conclusions can be drawn:

(1) Compared to the hot mix SMA-13 asphalt mixture, the low and intermediate-temperature cracking resistance of the hot recycled asphalt mixture deteriorated. The amplitude of the hot in-place recycled asphalt mixtures decreased more due to the higher percentage of RAP. In terms of intermediate-temperature cracking resistance, the hot in-place recycled mixture decreased by 22.1% and 28.6% (in terms of G*_f_* and FI, respectively), while the hot central plant recycled mixture decreased by 13.4% and 14.1%. In terms of low-temperature cracking resistance, the hot in-place recycled mixture decreased by 41.1% and 39.4% (in terms of *m*(*t*)/*S*(*t*) and *W_d_*/*W_s_*, respectively), while the hot central plant recycled mixture decreased by 36.4% and 6.7%.
(2) Basalt fiber improved the low and intermediate-temperature cracking resistance of recycled mixtures compared to lignin fiber. The enhancing amplitude in cracking resistance was greater for the hot in-place recycled mixture. In terms of intermediate-temperature cracking resistance, the hot in-place recycled mixture increased by 19.9% and 55.7% (in terms of G*_f_* and FI, respectively), while the hot central plant recycled mixture increased by 31.8% and 74.8%. In terms of low-temperature cracking resistance, the hot in-place recycled mixture decreased by 27.1% and 105.4% (in terms of *m*(*t*)/*S*(*t*) and *W_d_*/*W_s_*, respectively), while the hot central plant recycled mixture decreased by 33.0% and 45.6%. It was shown that basalt fibers could improve toughness and enhance energy dissipation.
(3) The results of the above four sets of cracking tests show that basalt fiber could effectively improve the cracking resistance of hot in-place recycled mixtures and hot central plant recycled mixtures, and the test results supplement the research on the application of basalt fiber in asphalt mixtures, which could provide an effective basis for the recycling of pavements.
(4) There were differences in the cracking resistance properties derived from different experimental methods, which may reflect different cracking mechanisms. Based on the correlation analysis, the results indicated that the trabecular bending test was more apt for evaluating the low-temperature cracking resistance of hot recycled asphalt mixtures, whereas the IDEAL-CT test was more suitable for assessing the intermediate-temperature cracking resistance of the mixtures.

## Figures and Tables

**Table 1:** Results for properties of extracted old asphalt.

Property	Old Asphalt	Requirements for SBS Modified Asphalt	Test Method JTG E20 [30]
Penetration (25 °C)/0.1 mm	39	50–80	T0604
Softening point/°C	69	>60	T0606
Ductility (5 °C)/cm	7.8	>30	T0605
Viscosity (135 °C)/Pa·s	2.33	≤3	T0613

**Table 2:** Mineral gradation of RAP.

Sieve Size/mm	16.0	13.2	9.5	4.75	2.36	1.18	0.6	0.3	0.15	0.075
Upper limit	100	100	75	34	26	24	20	16	15	12
Lower limit	100	90	50	20	15	14	12	10	9	8
Median	100	95	62.5	27	20.5	19	16	13	12	10
RAP	100.0	96.3	64.3	30.1	24.5	19.7	17.0	14.8	12.6	10.2

**Table 3:** Technical index of the rejuvenating agent RA-102.

Technical Index	RA-102	Requirements in JTG/T 5521-2019 [31]	Test Method JTG E20
Viscosity at 60 °C/cP	4000	— —	T0619
Flashpoint/°C	248	≥220	T0633
Saturated fraction content/%	25.6	≤30	T0618
Aromatic content/%	53	≥30	T0618
Viscosity ratio (RTFOT pre to post)	1.34	≤3	T0610
Mass change (%, RTFOT pre to post)	1.02	≤4%	T0603

**Table 4:** Technical index of basalt fiber.

Index	Results	Test Method
Diameter/μm	17	GB/T 7690.5 [33]
Length range/mm	3–9 mixed length	JT/T 776.1 [34]
Fracture strength/MPa	2430	GB/T 20310 [35]
Elongation at break/%	3.0	GB/T 20310
Modulus of elasticity/GPa	84.5	GB/T 20310
Heat resistance, retained fracture strength/%	94.8	JT/T 776.1
(Fe_2_O_3_ + FeO) content/%	9.68	GB/T 1549 [36]
Acidity factor	6.1	GB/T 1549

**Table 5:** Technical index of lignin fiber.

Index	Requirements	Results
Ash content/%	18 ± 5	19.3
PH Value	7.5 ± 1.0	7.7
Oil absorption	5 times the fiber quality	6.2

**Table 6:** Properties of recycled asphalt with different rejuvenating agent dosages.

Property	Rejuvenating Agent Dosage					New Asphalt	Test Method JTG E20
	0	4	6	8	10		
Penetration (0.1 mm, 25 °C)	39	60	68	74	78	71	T0604
Softening point (°C)	69	65	63	61	56	64	T0606
Ductility (cm, 5 °C)	7.8	22.4	28.6	31.4	34.6	48	T0605

**Table 7:** Design results of mix proportions.

Gradation Type	Fiber Type	Fiber Dosage/‰	Oil/Aggregate Ratio/%	Air Voids/%	Voids in Mineral Aggregate/%	Voids Filled with Asphalt/%	Marshall Stability/kN
Hot mixed SMA-13	Lignin fiber	3	6.0	3.8	17.2	74.8	8.4
	Basalt fiber (6 mm)	3	5.8	4.3	16.8	74.4	11.5
Hot central plant recycled SMA-13	Lignin fiber	3	6.1	4.4	17.5	74.9	10.3
	Basalt fiber (6 mm)	3	5.9	4.1	17.1	76.0.	12.1
Hot in-place recycled SMA-13	Lignin fiber	1	6.0	4.0	17.5	78.3	11.3
	Basalt fiber (6 mm)	3	6.0	4.1	17.5	76.9	12.5
Requirements	——	——	——	3~4.5	≥16.5	70~85	≥6

note: fiber content was calculated by the weight of recycled asphalt mixture.

**Table 8:** Results of leakage and flyaway tests.

Gradation Type	Fiber Type	Flyaway Losses/%	Leakage Losses/%
Hot mixed SMA-13	Lignin fiber	6.8	0.09
Hot mixed SMA-13	Basalt fiber (6 mm)	4.5	0.08
Hot central plant recycled SMA-13	Lignin fiber	7.3	0.08
Hot central plant recycled SMA-13	Basalt fiber (6 mm)	4.9	0.07
Hot in-place recycled SMA-13	Lignin fiber	7.9	0.09
Hot in-place recycled SMA-13	Basalt fiber (6 mm)	5.4	0.08
Requirements	——	≤15	≤0.1

**Table 9:** Pearson’s correlation coefficients and their degrees and interpretation.

Pearson’s correlation coefficients	**Size of Correlation**	**Interpretation**
	0.9 to 1.0 (−0.9 to −1.0)	Very high positive (negative) correlation
	0.7 to 0.9 (−0.7 to −0.9)	High positive (negative) correlation
	0.5 to 0.7 (−0.5 to −0.7)	Moderate positive (negative) correlation
	0.3 to 0.5 (−0.3 to −0.5)	Low positive (negative) correlation
	0.0 to 0.3 (−0.0 to −0.3)	Negligible correlation

## Data Availability

Data are contained within the article.
